# Supplementary material for: Educational climate of a pathology residency program at a tertiary care hospital
Source: PLoS One. 2024 May 23;19(5):e0303534. doi: 10.1371/journal.pone.0303534 (PMC11115291; doi:10.1371/journal.pone.0303534)
Supplement: S1 Table — This is the table for the test of normality that was conducted to confirm if data was normally distributed. (DOCX) [file pone.0303534.s002.docx]

**S1 Table. Tests of Normality Kolmogorov-Smirnov**

|  | Statistics | df | Sig. |
| --- | --- | --- | --- |
| Total final | .159 | 18 | .200* |

* This is a lower bound of the true significance.

a Lilliefors Significance Correction
