# Supplementary material for: Educational climate of a pathology residency program at a tertiary care hospital
Source: PLoS One. 2024 May 23;19(5):e0303534. doi: 10.1371/journal.pone.0303534 (PMC11115291; doi:10.1371/journal.pone.0303534)
Supplement: Attachment — Submitted filename: Responses to Reviewers.docx [file pone.0303534.s003.docx]

Reviewer #1: Overall Review:
Minor recommendations:
- In the discussion, expand on possible reasons for the lower feedback scores and differences by training year. Compare to any similar findings in the literature.
- Incomplete phrase (Negative perceptions about supervision have been observed in a study by?)

I have included possible reasons for variations in feedback scores in lines 174-183.

I have addressed the issue of incomplete phrases.

- Conclusion could be strengthened by providing specific recommendations based on your findings, beyond just suggesting further study of the factors influencing scores.

I have provided recommendations to address low feedback scores through implementation of interventions based on conceptual models. The exact sentences are in the discussion section in line 212-225 and lines 258-268.

- Carefully proofread for any typos, grammar issues, or awkward wording.

I have tried to address this issue to the best of my knowledge through re-reading multiple times.

- Make sure references are in correct journal format.

I have followed the PLOS ONE template now.

- Consider adding any limitations of the study design or sample.

I have included limitations now as a part of discussion.

Here are some minor language/writing issues I identified:
Some awkward phrasing, such as: "Despite the prevalence of positive perceptions with varying degrees of magnitude in different populations, prevalence of negative perceptions has also been observed in some studies." This could be smoothed out and clarified.

I have re-phrased this sentence. I have almost re-written most of the discussion section by considering the recommendations of one of the reviewer.

Typos - "infers" should be "implies", wellbeing of PGRs (extra spaces) etc. Carefully proofread.

I have replaced “infers” with “implies”, removed extra spaces before wellbeing of PGRs.

Need to be consistent with abbreviations. For example, "post-graduate" is written out sometimes and abbreviated "PG" other times. Pick one format.

I have now written post-graduate (PG) in the beginning and then followed with PG throughout the paper.

Uses words that sound too casual/conversational in academic writing like "bulk" and "infers"

Addressed.

Overall, the writing is pretty clear and understandable. Just needs minor editing for typos, consistency, and using more formal academic style. The paper would benefit from careful proofreading and having someone else review the writing. But I did not see any major scientific, methodological, or statistical issues from my review.

Reviewer #2: The idea is interesting and worth exploring. There are a few things which require further clarification.

Firstly, you need to expand on the groups which you compared between (on what basis were these groups created?).

I have now explained in the manuscript that the pathology residency is a 5-year program. Participants in the last three years were comparatively fewer in number. Therefore, I have condensed participants from 5 years of training into three groups for the purpose of study. Group 1 is less than 2 which means years 1, Group 2 is equal to 2 which means year 2, and Group 3 which mean more than 2 is equal to trainees in years 3, 4 and 5.

Secondly, it would be interesting to see your interpretation for the differences in perceptions and experiences based on your context.

I have included the interpretation for the differences in the opening paragraph of the Discussion section.

I would also be interested to learn of the impact this study has on your program (any policy changes?).

I believe the differences in the perceptions and experiences relate to the different challenges associated with the milestones to be achieved at each level. I have included that in the manuscript now. Even though policy making has not been updated yet, this evidence can be used to advocate for the needed changes through updated policy. I have made the adjustments accordingly in lines 266-267.

Lastly, there is some use of informal language, a few grammatical errors, and some missing information (specifically the name of a study is missing which was alluded to in the discussion).

I have tried to replace the names of authors and years with descriptions about the studies to which they are referred.

Reviewer #3: The article is well written. Here are a few comments:

P9, HCP: what is this abbreviation stans for?

HCP stands for “Health Care Professionals”. I have included it in the manuscript as well.
P10, inadequate in preparing residents to play their significant role: What is their significant role, could you be more specific?

I have addressed it in the manuscript. This phrase was awkwardly written. I have re-phrased it. The lines are now as: Pathology graduates and employers agree that existing pathology residency training remains inadequate in preparing the residents who are competent in clinical chemistry during their professional careers primarily due to less value placed by residents on learning clinical chemistry and lack of directors who supervise and manage labs of clinical chemistry [18].

P 11, results, first two lines: Review numbers (15: young, 15: >30)

I have edited this part. It meant a large majority of participants (15) were either less than or equal to 30 years of age, and a smaller number (3) were older than 30 years.

P 11, were female: repetitive

Deleted repetitive words.

P 12, table, Peer collaboration: missing number in neutral

Addressed. It was 1 participant.

P 15, in a study by However: missing information
Coaching and assessment vs feedback:
In my understanding, coaching and assessment are part of feedback. You cannot coach without giving feedback. I find it difficult that the residents have positive perspective regarding coaching and assessment and at the same time they report the opposite in feedback!

I agree that coaching and assessment are part of the feedback process. However, on the D-RECT scale, they are measured as separate constructs. As the coaching and assessment scores are high, and feedback scores are low. I have mostly re-written the entire discussion part as per the recommendations of one of the reviewers to elaborate and focus more on the construct of feedback as this is an important finding that is consistent throughout literature and efforts need to be taken to promote feedback scores rather than just assessing the reasoning behind differences in these scores.

I also have concerns regarding the low number of residents particularly in pathology disciplines other than histopathology and predominantly female participants, which is acknowledged by the authors. I would advise to involve more numbers to be more representative.

I agree that the numbers of the study are not generalizable. However, this is the limitation of the study as it was only focused on pathology residency program in one point in time. As the response rate is 100%, there was no option to enroll any more residents at that time. I agree that these are alarming numbers. However, in a lower middle-income country like Pakistan, where availability and disbursement of funds is a major issue; Pathology residency program is at a disadvantaged end. Therefore, it may be considered as a pilot study if due to lack of generalizability issue, there is a publication concern. However, in future longitudinal study designs may be implemented to ensure larger sample sizes in residency programs that have fewer number of residents. Sub-specialties of pathology that have even fewer number of residents would require a separate publication to discuss low enrollment numbers in different sub-specialties of pathology.

Considering females as major representatives is alarming. However, considering the income prospects after pathology residency program in Pakistan, most of the males do not opt Pathology residency as a first choice. This is also dependent upon the lack of equipment needed to run the clinical chemistry labs. However, a separate comprehensive study would be needed to discuss these aspects.
