# Supplementary material for: Educational climate of a pathology residency program at a tertiary care hospital
Source: PLoS One. 2024 May 23;19(5):e0303534. doi: 10.1371/journal.pone.0303534 (PMC11115291; doi:10.1371/journal.pone.0303534)
Supplement: Attachment — Submitted filename: Response to Reviewers.docx [file pone.0303534.s004.docx]

In reviewing the comments by the reviewers, I only found the comments by Reviewer # 2. Therefore, responses to reviewer # 2 have been included in this document.

Additionally, comment by the Academic Editor was to deposit my laboratory protocol in [protocols.io](http://protocols.io/). Even though my study is now beyond the protocol stage, I have registered with the protocols.io and entered my abstract for this manuscript without the results section to reserve the doi for this manuscript. Therefore, the reserved doi for this manuscript is:

DOI: dx.doi.org/10.17504/protocols.io.n2bvj3wk5lk5/v1 (Private link for reviewers: https://www.protocols.io/private/E5424EF9C00211EEAABF0A58A9FEAC02 to be removed before publication.)

6. Review Comments to the Author

Please use the space provided to explain your answers to the questions above. You may also include additional comments for the author, including concerns about dual publication, research ethics, or publication ethics. (Please upload your review as an attachment if it exceeds 20,000 characters)

Reviewer #1: (No Response)

Reviewer #2: Thank you for addressing my previous comments. There are, however, still a number of grammatical and typographical errors and use of informal language (e.g. it's [line 66], Study was conducted (instead of the study) [line103], writing the word less than and also adding he symbol [line 171]. I believe you need to thoroughly edit the manuscript.

Thank you for your comprehensive feedback. First, I addressed these specific issues in lines 66, 103, and 171. Then, after making adjustments to the content for the other feedback points especially with regards to discussion section, I sent the finalized manuscript to an international professional editor for editing the manuscript in accordance with the standardized English language to prevent any grammatical and typographical errors and use of informal language.

One of the biggest issues I have now with the review is the discussion. The originally submitted discussion was more specific and offered a rich argument. The revised discussion was too general and at times very difficult to follow. I believe this generalization took away from the strength of the original discussion.

Two of the other reviewers had the issue that the discussion included more content on all domains of D-RECT rather than focusing on the statistically significant findings and their implications. Therefore, considering your comment about the discussion, I have included the same discussion patterns and detailed description of each domain of D-RECT as was in the first draft that you believed added to the beauty of the discussion. In addition, I have retained the content that was requested by the other reviewers with respect to feedback and variations in EC scores by years of training. Therefore, I have tried to the best of my abilities to preserve the feedback of all three reviewers in the final discussion section of the current manuscript version.

There were also some repetitive points made (e.g. the point about positive perception except in feedback [lines 228-232] and the point about the inability to enroll additional participants).

I have deleted all the repetitive points. Yes, there were specially at two points where the points were repeated about the need for longitudinal study to enroll larger sample size corresponding to the lower sample size in the current study that was based on cross-sectional study design. The point is that the study was completed at one point in time. It was not possible to enroll any more residents at that time as the response rate was 100%, and the new residents would be hired for the residency program in the corresponding year. The researcher had to complete the study in one year as this study was part of the thesis of a Masters’ degree. It does not mean any new additional participants cannot be enrolled. However, for that purpose a longitudinal study would be more feasible.

In some qualitative studies with executive leaders the number of participants is much less as compared to the sample in the general population. The same principle applies here that as we only targeted pathology residents in one tertiary care hospital, it was not possible to enroll any more participants. In other words, there were no more eligible participants as our study was not multidisciplinary. It was only aimed at analyzing the educational climate within the pathology residency program context.

Reviewer #3: (No Response)
